# Association of Bcr-Abl Tyrosine Kinase Inhibitors With Hepatitis B Virus Reactivation Requiring Antiviral Treatment in Taiwan

**DOI:** 10.1001/jamanetworkopen.2021.4132

**Published:** 2021-04-06

**Authors:** Ling-Yi Wang, Sung-Chao Chu, Yin Lo, Yen-Yun Yang, K. Arnold Chan

**Affiliations:** 1 Epidemiology and Biostatistics Consulting Center, Department of Medical Research, Buddhist Tzu Chi General Hospital, Hualien, Taiwan; 2 Department of Pharmacology, School of Medicine, College of Medicine, Tzu Chi University, Hualien, Taiwan; 3 Department of Pharmacy, Buddhist Tzu Chi General Hospital, Hualien, Taiwan; 4 Department of Hematology and Oncology, Buddhist Tzu Chi General Hospital, Hualien, Taiwan; 5 Department of Pharmacy, National Taiwan University Hospital; 6 Health Data Research Center, National Taiwan University; 7 Department of Medical Research, National Taiwan University Hospital

## Abstract

**Importance:**

The US Food and Drug Administration (FDA) highlighted the potential risk of hepatitis B reactivation that was associated with Bcr-Abl tyrosine kinase inhibitor (TKI) treatment and has required updated product labels.

**Objective:**

To examine the association between hepatitis B flare and exposure to Bcr-Abl TKIs compared with non–Bcr-Abl TKIs.

**Design, Setting, and Participants:**

This nested case-control study included patients who entered a hepatitis B carrier cohort in Taiwan after January 1, 2005. Patients who received their first antiviral agents for hepatitis B flare for more than 28 days after the cohort entry date were included as case patients. For each case, a corresponding risk set was formed that included all eligible patients in the study cohort who had the same age (within 1 year), same sex, and were at risk of developing hepatitis B flare at the case date. As many as 10 control patients were randomly selected from the risk set for each case patient. TKIs were evaluated before the hepatitis B flare for case patients and before the corresponding index date for control patients. Data were collected from the Taiwan National Health Insurance research database from January 2000 to 2015. Data analysis was conducted from January to June 2019.

**Exposure:**

Use of Bcr-AbL TKIs.

**Main Outcomes and Measures:**

Conditional logistic regression was used to estimate the rate ratio for the association between hepatitis B flare and exposure to Bcr-Abl TKIs compared with non–Bcr-Abl TKIs.

**Results:**

Among 698 342 patients who carried incident hepatitis B virus, 66 702 patients with hepatitis B flare that required antiviral treatment (47 492 [71.2%] men; mean [SD] age at index date, 50.2 [13.8] years) were included as case patients, and 666 989 age and sex–matched patients (474 903 [71.2%] men; mean [SD] age, 50.2 [13.8] years) were included as control patients. Analysis revealed that Bcr-Abl TKI use during the previous 90 days was independently associated with a 56% higher risk of hepatitis B flare (adjusted rate ratio [aRR], 1.56; 95% CI, 1.11-2.20), and the aRR increased to 1.66 (95% CI, 1.20-2.28) for Bcr-Abl TKI use during the previous 365 days. Use of Bcr-AbL TKIs during the previous 60 days was associated with a significantly increased risk of flare among women (aRR, 3.20; 95% CI, 1.70-6.03) but not among men (aRR, 1.14; 95% CI, 0.72-1.81).

**Conclusions and Relevance:**

These findings suggest that sex-specific strategies may be needed to monitor for hepatitis B reactivation among patients receiving Bcr-Abl TKIs.

## Key Points

**Question:**

Is the use of Bcr-Abl tyrosine kinase inhibitors (TKIs) associated with subsequent hepatitis B virus reactivation?

**Findings:**

In this nationwide nested case-control study with 733 691 individuals carrying the hepatitis B virus, Bcr-Abl TKIs were associated with overt HBV reactivation that required antiviral treatment. Women receiving Bcr-Abl TKIs had higher odds of reactivation than men.

**Meaning:**

To our knowledge, this was the first population-based study that found that Bcr-Abl TKIs were associated with increased risk of HBV reactivation.

## Teaser

This case-control study examines the association between hepatitis B flare and exposure to Bcr-Abl tyrosine kinase inhibitors (TKIs) compared with non–Bcr-Abl TKIs.

## Introduction

Drug-induced liver injury is an infrequent but challenging complication of drug therapy,^1^ and drug-induced hepatitis B virus (HBV) reactivation is particularly important in an HBV endemic area. The highest prevalence of HBV infections is in the western Pacific region (6.2% of adults) and Africa (6.1% of adults).^2^ In 1984, Taiwan implemented vaccination programs for infants and high-risk groups, although approximately 15% to 20% of adults still carry HBV.^3^

In patients who receive immunosuppressants, HBV reactivation is a well-recognized complication, especially among those who receive high-dose or long-term use of steroids,^4,5^ biologic drugs (eg, rituximab),^6^ and chemotherapy.^7,8,9^ In this setting, HBV reactivation–related hepatitis or hepatitis B flare is a clinical event that involves an abrupt increase in alanine aminotransferase (ALT) concentrations (to >2-5 times the upper reference limit), and its presentation may vary from asymptomatic to overt acute hepatitis, including hepatic failure.^10^ Routine HBV screening is recommended before treatment using these medications, and strategies for preventing and treating HBV reactivation are well documented.^5,6,9^ However, there is less information regarding the risks of HBV reactivation associated with targeted therapies, including tyrosine kinase inhibitors (TKIs).

Small-molecule inhibitors that target Bcr-Abl tyrosine kinase (Bcr-Abl TKIs, including imatinib, nilotinib, and dasatinib) are standard treatment for chronic myeloid leukemia (CML) and gastrointestinal stromal tumors (GISTs). However, several case reports and small-scale studies have suggested that Bcr-Abl TKIs may be associated with an increased risk of HBV reactivation.^11,12,13,14,15,16,17,18^ Furthermore, in 2016, the US Food and Drug Administration (FDA) highlighted the potential risk of HBV reactivation associated with Bcr-Abl TKI treatment and required updated product labels.^19^ Several in vitro studies have indicated that Bcr-Abl TKIs exert inhibitory effects on T cells,^20^ and it remains unclear whether this adverse effect is observed in patients, as immune-related T cell inhibition is a risk factor for HBV reactivation. In this study, we aimed to evaluate whether Bcr-Abl TKI use was associated with hepatitis B flare in Taiwan, which is an HBV endemic area.

## Methods

This study followed the Strengthening the Reporting of Observational Studies in Epidemiology (STROBE) reporting guideline for case-control studies.^21^ The National Taiwan University Hospital research ethics committee exempted the present study from review and waived the requirement for informed consent because of the use of deidentified data.

### Data Source

Taiwan National Health Insurance (NHI) was launched in 1995 as a government-sponsored single-payer insurance system. All residents living in Taiwan for longer than 6 months are required to enroll, and approximately 23 million beneficiaries were registered with the NHI by the end of 2016 (a 99.5% coverage rate). The NHI data include outpatient clinic and hospitalization service claims, which are processed electronically. The NHI system provides a complete diagnostic history (using *International Classification of Disease, 9th Revision, Clinical Modification* [*ICD-9-CM*] codes), prescriptions, procedures, and special examinations associated with covered claims, which are linked to unique personal identifier numbers. All data are deidentified before being made available to researchers.

### Study Population

#### Definition of HBV Carrier Cohort for the Case-Control Study

We defined individuals as carrying HBV if they fulfilled 1 of 3 criteria: (1) a diagnosis of HBV infection (*ICD-9-CM* codes 070.2, 0.70.3, or V02.61) twice within 1 year; (2) an HBV infection diagnosis and 1 measurement of the HBV e antigen or antibodies to HBV e with an interval of 30 days or less between the diagnosis and measurement dates (or vice versa); and (3) hospitalization with a primary diagnosis of HBV infection. Patients who fulfilled criterion 1 or 2 had their cohort entry date defined as the earliest diagnosis or measurement date. Patients who fulfilled criterion 3 had their cohort entry date defined as the hospital admission date. Data were evaluated for 2000 to 2015, and patients were considered eligible if their cohort entry date was after January 1, 2005. The earliest event date was used as the cohort entry date for participants with multiple eligible events. The exclusion criteria were being younger than 18 years at the cohort entry date and/or having a diagnosis of hepatitis C (*ICD-9-CM* codes 070.54, 070.70, or V02.62) before the cohort entry date.

#### Identification of Case Patients

The primary outcome was defined as hepatitis B flare (a severe type of HBV reactivation), as data regarding HBV DNA, bilirubin concentration, prothrombin time, and international normalized ratio were not available to clinically substantiate HBV reactivation.^10^ We defined case patients as those who received their first antiviral agents for hepatitis B flare for more than 28 days after the cohort entry date. In those instances, the first antiviral prescription date was treated as the case date. Antiviral agents were conditionally covered by the NHI for chronic patients who carried HBV with (1) a more than 5-fold increase in ALT concentrations or (2) a 2- to 5-fold increase in ALT concentrations plus HBV DNA values of 20 000 IU/mL or greater.

Five antiviral agents were considered in this study: lamivudine, tenofovir, adefovir dipivoxil, entecavir, and telbivudine. The following cytotoxic chemotherapy agents were also evaluated because of their documented risk of HBV reactivation^8^: 5-fluorouracil, capecitabine, carboplatin, cytarabine, cyclophosphamide, docetaxel, daunorubicin, epirubicin, etoposide, gemcitabine, irinotecan, idarubicin, methotrexate, oxaliplatin, doxorubicin, paclitaxel, and vinorelbine. We excluded patients who received these chemotherapy drugs or had NHI prescription codes that were associated with chemotherapy administered in a clinic within 30 days after the case date to ensure that the antiviral agents were prescribed for treating a hepatitis B flare, rather than for prophylactic purposes. The Anatomical Therapeutic Chemical (ATC) codes for the drugs of interest and the NHI procedure codes are listed in the eTable in the Supplement.

#### Identification of Control Patients

For each case, we formed a corresponding risk set that included all eligible patients in the study cohort who had the same age (within 1 year), same sex, and were at risk of developing hepatitis B flare at the case date. As many as 10 control patients were randomly selected from each case’s risk set. For the analyses, all control patients in a risk set were assigned the same index date as the case date for the corresponding case.

#### Exposure Ascertainment and Covariate Adjustment

The exposure of interest was defined as the use of 3 Bcr-Abl TKIs that were covered in Taiwan during the study period: imatinib, dasatinib, and nilotinib. Patients who received Bcr-Abl TKIs were identified based on a prescription within 60 days, 90 days, 120 days, 180 days, and 365 days before the index date, and all other patients were considered not to have used Bcr-Abl TKIs. Potential confounders included prior use of immunosuppressants (ie, prednisolone, methylprednisolone, dexamethasone, hydrocortisone, everolimus, sirolimus, tacrolimus, azathioprine, cyclosporine, mycophenolate), cytotoxic chemotherapy or rituximab (ATC codes for these drugs are listed in the eTable in the Supplement), and various diseases and conditions. The diseases and conditions included allogeneic or autologous transplantation for hematological malignant neoplasm (<1 year before the index date), alcoholic cirrhosis, biliary cirrhosis, alcoholic liver disease (≥2 diagnoses in the 6 months before the index date), malignant neoplasms that might be treated using Bcr-Abl TKIs (eg, CML, monocytic leukemia, acute lymphoblastic leukemia, acute myeloid leukemia, GIST), and other cancer types. If a patient had multiple cancer diagnoses, the cancer type was determined in the following order: acute lymphoblastic leukemia, acute myeloid leukemia, monocytic leukemia, CML, GIST, and then other solid cancers. The presence of hematological malignant neoplasms was determined based on at least 2 diagnoses in the 6 months before the index date.

### Statistical Analysis

Descriptive statistics were used to summarize the characteristics of the case patients and matched control patients. The primary analysis evaluated the association between Bcr-Abl TKI use and hepatitis B flare, based on a history of Bcr-Abl TKI use during the 60-day, 90-day, 120-day, 180-day, and 365-day periods before the index date. Sensitivity analysis was performed by excluding case patients with a record of chemotherapy during the 30 days before or after the index date. Conditional logistic regression analysis was used to estimate the adjusted rate ratios (aRRs) and 95% CIs for hepatitis B flare during the 6 months or 12 months before the index date according to Bcr-Abl TKI use. We also performed stratified analyses according to age (≥50 years vs <50 years) and sex. All analyses were performed using SAS software version 9.4 (SAS Institute). The analysis was performed from January to June 2019. No statistical tests were performed, so no prespecified level of significance was set.

## Results

Among 698 342 patients who carried incident hepatitis B virus identified from Taiwan NHI from 2005 through 2015, there were 66 702 patients with cases of hepatitis B flare that required antiviral treatment (47 492 [71.2%] men; mean [SD] age at index date, 50.2 [13.8] years), and 666 989 age and sex–matched control patients (474 903 [71.2%] men; mean [SD] age, 50.2 [13.8] years) (Figure). More individuals included as case patients received Bcr-Abl TKIs before the index date than those included as control patients. During the 60 days before the index date, 58 case patients (0.09%) used Bcr-Abl TKIs vs 161 control patients (0.02%). The prevalence of malignant neoplasms was relatively low for both case patients and matched control patients, although case patients were more likely to have hematological malignant neoplasms and other cancers. Cirrhosis and transplantation for hematological malignant neoplasms were also more prevalent among the case patients, and the case patients were also more likely to be exposed to medications with an increased risk of HBV reactivation (Table 1). The conditional logistic regression analysis (adjusted for all potential confounders) revealed that Bcr-Abl TKI use during the 90 days before the index date was associated with a 56% higher risk of hepatitis B flare (aRR, 1.56; 95% CI, 1.11-2.20) (Table 2). This association with an increased risk of hepatitis B flare was significant for the 120-day, 180-day, and 365-day assessment windows, with the largest apparent association observed for Bcr-Abl TKI use during the 365 days before the index date (aRR, 1.66; 95% CI, 1.20-2.28). The sensitivity analyses revealed similar results for Bcr-Abl TKI use during the 60 days before the index date (aRR, 1.49; 95% CI, 1.04-2.14) and during the 365 days before the index date (aRR, 1.69; 95% CI, 1.22-2.34).

**Figure.**
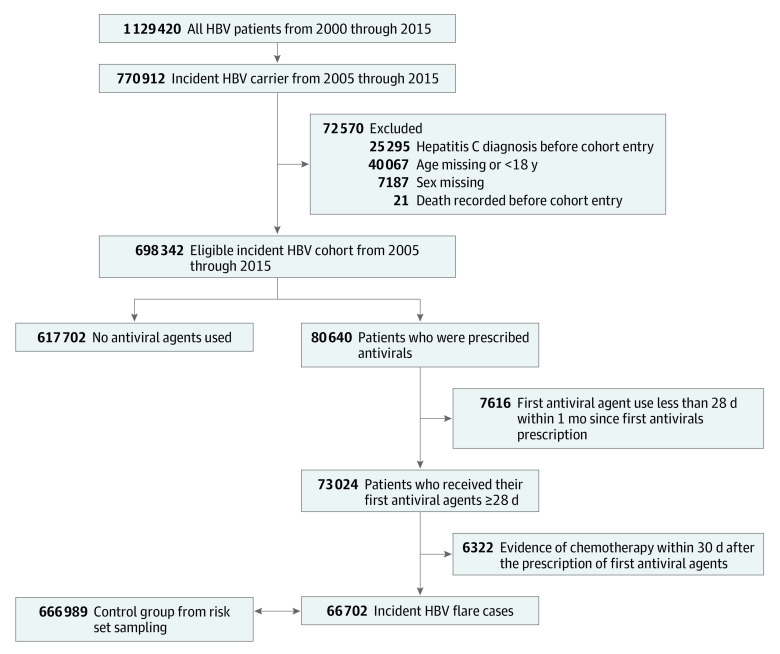
Flowchart of Case and Matched Control Selection HBV indicates hepatitis B virus.

**Table 1.** Characteristics of Case Patients With Hepatitis B Flare and Matched Control Patients Among a Cohort of Individuals Carrying Hepatitis B Virus in Taiwan

Characteristic	No. (%)	
	Case patients (n = 66 702)	Matched control patients (n = 666 989)
Age, mean (SD), y	50.18 (13.83)	50.18 (13.83)
Men	47 492 (71.20)	474 903 (71.20)
Women	19 210 (28.80)	192 086 (28.80)
Bcr-Abl TKI used before index date^a^		
60 d	58 (0.09)	161 (0.02)
90 d	64 (0.10)	165 (0.02)
180 d	69 (0.10)	172 (0.03)
365 d	73 (0.11)	180 (0.03)
Cancer history 6 mo prior to the index date		
Chronic myeloid leukemia and monocytic leukemia^b^	26 (0.04)	106 (0.02)
Acute leukemia		
Myeloid	85 (0.13)	175 (0.03)
Lymphoid	79 (0.12)	138 (0.02)
Soft-tissue tumor	1022 (1.53)	2944 (0.44)
Other cancer	18 121 (27.17)	50 628 (7.59)
No diagnosis	47 369 (71.02)	612 998 (91.91)
Other comorbidities		
Cirrhosis and other liver disease^c^	1152 (1.73)	3516 (0.53)
Transplantation for hematologic malignant neoplasms^d^	34 (0.05)	63 (0.01)
Medications as a known risk factor hepatitis B flare		
Immunosuppressants^e^	4172 (6.25)	16 031 (2.40)
Cytotoxic chemotherapy^f^	1844 (2.76)	4347 (0.65)
Rituximab	89 (0.13)	181 (0.03)

Abbreviation: TKI, tyrosine kinase inhibitor.

^a^ The number of case and control patients receiving Bcr-Abl TKIs at 120 days was too close to its adjacent category; therefore, the data could not be released.

^b^ Number of cases of monocystic leukemia was smaller than the threshold for data release; therefore, the number of cases of monocystic leukemia was combined with those of chronic myeloid leukemia.

^c^ Alcohol cirrhosis, biliary cirrhosis, or alcoholic liver disease.

^d^ Transplantation included allogenic or autologous. Transplantation history was assessed 1 year prior to the index date, and at least 1 or more record of transplantation was regarded as having transplantation history.

^e^ Immunosuppressants include systemic steroids (prednisolone, methylprednisolone, dexamethasone, hydrocortisone) and other immunosuppressants (everolimus, sirolimus, tacrolimus, azathioprine, cyclosporin, mycophenolate). Use 180 days prior to index date with cumulative use of at least 28 days was classified as immunosuppressant users.

^f^ Cytotoxic chemotherapy included 5-fluorouracil, gemcitabine, capecitabine, methotrexate, oxaliplatin, carboplatin, cyclophosphamide, doxorubicin, epirubicin, irinotecan, etoposide, paclitaxel, docetaxel, vinorelbine, cytarabine, daunorubicin, idarubicin, or other record of having chemotherapy in the clinics.

**Table 2.** Association Between Bcr-Abl TKI Use and Incident Hepatitis B Flare

TKI use prior to index date, d	Rate ratio (95% CI)^a^	
	Primary^b^	Sensitivity^c^
60	1.41 (0.99-2.01)	1.49 (1.04-2.14)
90	1.56 (1.11-2.20)	1.62 (1.14-2.30)
120	1.64 (1.17-2.30)	1.69 (1.20-2.38)
180	1.60 (1.15-2.23)	1.67 (1.19-2.35)
365	1.66 (1.20-2.28)	1.69 (1.22-2.34)

Abbreviation: TKI, tyrosine kinase inhibitors.

^a^ Rate ratios were estimated by the conditional logistic regression. Covariates considered in the models were Bcr-Abl TKI use, cancer history in 6 months prior to the index date, cirrhosis, transplantation for hematologic malignant neoplasms, and other medications that are well known for their hepatitis B flare HBV reactivation risk (ie, immunosuppressants, cytotoxic chemotherapy, and rituximab).

^b^ In the primary analysis, case patients were those who received their first antiviral agents for more than 28 days with no evidence of receiving chemotherapy within 30 days after index date.

^c^ In the sensitivity analysis, case patients were those who received their first antiviral agents for more than 28 days with no evidence of receiving chemotherapy within 30 days before and after index date.

Stratified analyses revealed sex-specific differences in the risk of hepatitis B flare associated with Bcr-Abl TKI use (Table 3). Among women, Bcr-Abl TKI use during the 60 days before the index date was associated with a 3-fold higher risk of hepatitis B flare (aRR, 3.20; 95% CI, 1.70-6.03), and this risk was even greater when we considered Bcr-Abl TKI use during the 365 days before the index date (aRR, 3.61; 95% CI, 2.03-6.40). However, among men, the association between Bcr-Abl TKI use and the risk of hepatitis B flare during the 60 days before the index date (aRR, 1.14; 95% CI, 0.72-1.81) or during the 365 days before the index date (aRR, 1.29; 95% CI, 0.84-1.98) was not observed (Table 3). Among those carrying HBV who were 50 years or older, the risk of hepatitis B flare was associated with Bcr-Abl TKI use during the 120-day, 180-day, and 365-day windows. However, these associations were not observed among those carrying HBV who were younger than 50 years (Table 3).

**Table 3.** Association Between Bcr-Abl TKI Use and Incident Hepatitis B Flare, Stratified by Sex and Age

TKI use prior to index date, d	Rate ratio (95% CI)^a^			
	Male patients	Female patients	Patients by age, y	
			<50	≥50
60	1.14 (0.72-1.81)	3.20 (1.70-6.03)	1.38 (0.63-3.02)	1.36 (0.91-2.04)
90	1.31 (0.84-2.05)	3.44 (1.86-6.39)	1.82 (0.86-3.85)	1.44 (0.97-2.14)
120	1.32 (0.85-2.06)	3.74 (2.04-6.86)	1.72 (0.82-3.60)	1.56 (1.06-2.30)
180	1.30 (0.84-2.02)	3.63 (2.00-6.57)	1.87 (0.91-3.86)	1.49 (1.02-2.18)
365	1.29 (0.84-1.98)	3.61 (2.03-6.40)	1.81 (0.88-3.73)	1.57 (1.09-2.26)

Abbreviation: TKI, tyrosine kinase inhibitors.

^a^ Rate ratios were estimated by the conditional logistic regression. Covariates considered in the models were Bcr-Abl TKI use, cancer history in 6 months prior to the index date, cirrhosis, transplantation for the hematologic malignancies, and other medications that are well known for their hepatitis B flare or HBV reactivation risk (ie, immunosuppressants, cytotoxic chemotherapy, and rituximab). In the primary analysis, case patients were those who received their first antiviral agent for more than 28 days with no evidence of receiving chemotherapy within 30 days after index date.

## Discussion

To our knowledge, this is the first population-based cohort study to evaluate the association between Bcr-Abl TKI use and risk of hepatitis B flare. After adjusting for a broad range of potential confounding factors, we found that Bcr-Abl TKI use was associated with an increased risk of hepatitis B flare in Taiwan. The risk of hepatitis B flare also increased with longer windows for Bcr-Abl TKI exposure, suggesting a cumulative effect. Moreover, the risk of Bcr-Abl TKI–related hepatitis B flare was more pronounced among women.

In 2016, the US FDA required that the product labels for imatinib, dasatinib, and nilotinib be updated to highlight concerns regarding the risk of HBV reactivation.^19^ However, those warnings were based on anecdotal case reports, which could not confirm a causal relationship between Bcr-Abl TKI use and HBV reactivation. The NHI data do not include laboratory findings that are needed to clinically identify HBV reactivation (eg, HBV e antigen status, evidence of HBV DNA replication, or an abrupt increase in liver enzyme concentrations). Therefore, the present study defined hepatitis B flare based on the prescription of 5 antiviral agents (entecavir, lamivudine, adefovir, telbivudine, or tenofovir), which are covered by the NHI for treatment of HBV reactivation. The present study could possibly include individuals with HBV reactivation who did not receive treatment as control patients, and such misclassified control patients would bias the findings toward the null association, resulting in an underestimation of the true strength of the association between Bcr-Abl TKI use and hepatitis B flare. Furthermore, the association between Bcr-Abl TKI use and hepatitis B flare was consistently observed across different exposure periods in the sensitivity analysis. Moreover, the nested case-control design efficiently estimates the unbiased RR from the cohort.^22^

Patients receiving Bcr-Abl TKIs are thought to have a moderate risk (1%-10%) of HBV reactivation,^6^ and the existing case reports have suggested that HBV reactivation tends to occur during the 3 to 6 months after starting Bcr-Abl TKI treatment (range, 1 week to 53 months).^11,12,13,15,16,17^ However, there is no consensus regarding strategies for the identification or prophylactic treatment of HBV reactivation in patients who are receiving TKIs. Nevertheless, diligent surveillance has traditionally been recommended for all patients who are receiving TKIs.^23^ In Taiwan, the latest prescribing information for imatinib, nilotinib, and dasatinib also highlights the potential risk of HBV reactivation, and clinicians “should screen for HBV before starting these TKIs and should closely monitor for clinical and laboratory signs of HBV reactivation”.^24,25,26^

Hepatitis B flare rarely occurs in individuals carrying HBV who are asymptomatic, although it is a well-recognized phenomenon among those carrying HBV who are receiving immunosuppressive or biologic treatment.^6^ Furthermore, hepatitis B flare is most commonly reported among patients who are receiving chemotherapy for hematological malignant neoplasms.^9,27,28^ A review of HBV reactivation after TKI use has suggested that off-target immunological effects likely play an important role.^29^ Moreover, a recent review of risk factors for HBV reactivation in patients with hematological disorders^28^ identified several independent risk factors: male sex, age 50 years or older, viral factors, and certain medications. We considered most of these risk factors, with the exception of laboratory parameters, in our regression model, which revealed consistent positive associations with hepatitis B flare. The age-stratified analyses revealed a significantly increased risk of hepatitis B flare (an increase of approximately 50%) associated with Bcr-Abl TKI use for individuals who were 50 years or older during the 120-day, 180-day, and 365-day windows. Similar magnitude aRRs were observed among individuals who were younger than 50 years, although the 95% CIs all included 1 (Table 3).

The 3 Bcr-Abl TKIs (imatinib, nilotinib, and dasatinib) are standard treatment for CML and GIST. However, the immunosuppressive properties of these drugs have also attracted attention regarding their potential ability to trigger HBV reactivation.^11,12,13,14,15,16,17,18^ Furthermore, several case reports have supported the hypothesis of HBV reactivation during the immune-restoration stage, given that the hepatic flare was observed after the patients achieved complete molecular or cytogenetic responses.^11,12,13,15,16,17^ The Bcr-Abl TKIs may target different pathways for immune restoration,^15^ as lymphocytopenic status is typically influenced by imatinib,^11^ which generally inhibits T-cell proliferation and activation.^30,31^ Patients with CML treated with dasatinib have impaired immune surveillance, and leukemic-specific T cells are functionally exhausted or anergic.^32^ Thus, although the mechanisms for TKI-induced hepatitis B flare remain unclear, various tyrosine kinase–mediated signaling pathways are involved in the immune activation and proliferation of lymphocytes, and blockade of these pathways may suppress the immune control of HBV and lead to reactivation.^28^

Female patients have a 1.5- to 1.7-fold greater risk of developing an adverse drug reaction compared with male patients.^33,34^ The present study also highlighted the immune-stimulating effects of estrogen, which leads to a more profound response to virus-related hepatitis in women.^35,36^ We also observed that women who received Bcr-Abl TKI had an approximately 3-fold higher risk of hepatitis B flare than women who did not receive TKIs, and this association was not observed among men (Table 3). Women may also be more susceptible than men to drug-related acute liver failure and autoimmune hepatitis^37^ given that the liver has been described as a sexually dimorphic organ that responds to sex hormones by expressing androgen and estrogen receptors. This may help to explain the sex-based differences in immune response and xenobiotic metabolism (based on pharmacokinetic and pharmacodynamic parameters). Sex hormones regulating the immune response and inflammation may also influence hepatitis-related adverse drug reactions, based on women having higher levels of hepatic pro-inflammatory cytokines, greater antibody production, and more severe hepatitis.^35^ The American Gastroenterological Association guidelines for preventing HBV reactivation during immunosuppressive therapy provide a weak recommendation to perform antiviral prophylaxis or monitoring for patients at moderate risk who are undergoing TKI therapy (based on moderate-quality evidence).^5^ Our findings support performing antiviral prophylaxis over monitoring among patients who are receiving Bcr-Abl TKIs, especially for women carrying HBV.

### Limitations

The study has limitations. First, patients receiving Bcr-Abl TKIs will typically undergo intensive liver function monitoring, and the more careful clinical examinations in these cases might be more likely to identify asymptomatic HBV reactivation (vs among patients who do not receive Bcr-Abl TKIs). Nevertheless, we performed case ascertainment based on antiviral use for more than 28 days, which would suggest more severe and apparent clinical symptoms that required treatment, and surveillance bias might not be a major concern. Second, our ascertainment criteria for HBV reactivation were only applicable to overt HBV reactivation or hepatitis B flare cases that required antiviral treatment. Third, without information about the antibody HB core antigen, we could not assess whether HBV flare-up risk differed between those who carried HBV (ie, patients who tested positive for HB s antigens) and those with latent HBV (ie, patients who tested negative for HB s antigens and positive for HB c antibodies).^6^ Fourth, the limited sample size for some drugs precluded individual analyses of the 3 Bcr-Abl TKIs, and ponatinib (a fourth Bcr-Abl TKI) was not covered by the NHI during the study period, although we expect that a class effect would have been observed for all 4 TKIs.^38^ Fifth, the NHI data did not include information regarding smoking habit, alcohol consumption, and body mass index, which precluded related analyses.

## Conclusions

This case-control study found that, in Taiwan, use of Bcr-Abl TKIs was associated with an increased risk of hepatitis B flare, especially among women. Prospective studies are needed to investigate the effectiveness of sex-specific prophylactic antiviral treatments or monitoring strategies for those carrying HBV who are about to receive Bcr-Abl TKIs.

## Notes

Supplement. **eTable.** Anatomical Therapeutic Chemical (ATC) Classification Codes for Drugs, Taiwan National Health Insurance Codes for Procedures of Interest, and *ICD-9-CM* Codes for Diseases of Interest
