# Supplementary material for: Association of Bcr-Abl Tyrosine Kinase Inhibitors With Hepatitis B Virus Reactivation Requiring Antiviral Treatment in Taiwan
Source: JAMA Netw Open. 2021 Apr 6;4(4):e214132. doi: 10.1001/jamanetworkopen.2021.4132 (PMC8025118; doi:10.1001/jamanetworkopen.2021.4132)
Supplement: Supplement. — eTable. Anatomical Therapeutic Chemical (ATC) Classification Codes for Drugs, Taiwan National Health Insurance Codes for Procedures of Interest, and ICD-9-CM Codes for Diseases of Interest [file jamanetwopen-e214132-s001.pdf]

Supplemental Online Content

Wang LY, Chu SC, Lo Y, Yang YY, Chan KA. Association of Bcr-Abl tyrosine kinase inhibitors with hepatitis B virus reactivation requiring antiviral treatment in Taiwan. *JAMA Netw Open*. 2021;4(4):e214132. doi:10.1001/jamanetworkopen.2021.4132

eTable. Anatomical Therapeutic Chemical (ATC) Classification Codes for Drugs, Taiwan National Health Insurance Codes for Procedures of Interest, and *ICD-9-CM* Codes for Diseases of Interest

This supplemental material has been provided by the authors to give readers additional information about their work.

© 2021 Wang LY et al. *JAMA Network Open.*

eTable. Anatomical Therapeutic Chemical (ATC) Classification Codes for Drugs, Taiwan National Health Insurance Codes for Procedures of Interest, and *ICD-9-CM* Codes for Diseases of Interest

Drug	ATC
lamivudine	J05AF05
tenofovir	J05AF07
adefovir dipivoxil	J05AF08
entecavir	J05AF10
telbivudine	J05AF11
imatinib	L01XE01
datatinib	L01XE06
nilotinib	L01XE08
5-fluorouracil	L01BC02, L01BC52
gemcitabine	L01BC05
capecitabine	L01BC06
methotrexate	L01BA01, L04AX03
oxaliplatin	L01XA03
carboplatin	L01XA02
cyclophosphamide	L01AA01
doxorubicin	L01DB01
epirubicin	L01DB03
irinotecan	L01XX19
etoposide	L01CB01
paclitaxel	L01CD01
docetaxel	L01CD02
vinorelbine	L01CA04
azathioprine	L04AX01
cyclosporin	L04AD01
mycophenolate	L04AA06
rituximab	L01XC02
cytarabine	L01BC01
daunorubicin	L01DB02
idarubicin	L01DB06
everolimus	L01XE10, L04AA18
sirolimus	L04AA10, L01XE09
tacrolimus	L04AD02
prednisolone	H02AB06
methylprednisolone	H02AB04
dexamethasone	H02AB02
hydrocortisone	H02AB09
**Procedure of interest**	**Taiwan National Health Insurance codes**
HBeAg measurement	14034C, 14035C, 27035B
Anti-Hbe measurement	14036C and 27036B

© 2021 Wang LY et al. *JAMA Network Open.*

Receiving chemotherapy in the clinics	37031B, 37032B, 37033B, 37038B,37039B, 37040B, 37041B
Autologous stem-cell transplantation	94202B, 94207B, 94202A
Allogeneic stem cell transplantation	94201B, 94206B, 94204B, 94201A, 48006C1
Diagnosis	International classification of Disease, 9th revision, Clinical Modification code
Cirrhosis	571.0, 571.1, 571.2, 571.3, 571.6
Chronic myeloid leukemia	205.1
Monocytic leukemia	206
Acute lymphoblastic leukemia	204
Acute myeloid leukemia	205
Gastrointestinal stromal tumor	238-239
Other cancers (except aforementioned hematologic disorders)	140-208

© 2021 Wang LY et al. *JAMA Network Open.*
